# Mean platelet volume as a translational biomarker for cardiovascular risk stratification with perspectives for medical education: a systematic review

**DOI:** 10.21542/gcsp.2026.12

**Published:** 2026-04-30

**Authors:** Diego Fernando López-Muñoz, Cristhian Camilo Velandia-Mosquera, Oscar Eduardo Cruz-Becerra, Daniela Andrea Agudelo, Esteban Pineda-Arias, Martha Liliana Girón-Girón

**Affiliations:** Faculty of Health Sciences, Department of Medicine, Health Sciences Research and Education Group–GIECSA. Unidad Central del Valle del Cauca. Tuluá, Valle del Cauca, Colombia

## Abstract

**Objective**:To evaluate the prognostic value of mean platelet volume (MPV) for cardiovascular risk stratification in settings with limited resources.

**Methods**: We conducted a comprehensive systematic review in accordance with the PRISMA 2020 guidelines (PROSPERO registration number: CRD420251156089). From January 2019 to December 2024, we searched five databases (PubMed, Scopus, Web of Science, SciELO and LILACS) for observational studies linking MPV to cardiovascular outcomes. The methodological quality of the studies was assessed using the STROBE criteria, and the certainty of the evidence was assessed using the GRADE approach.

**Results**: Thirteen studies involving 3,085 participants were included. Of these, 12 (92.3%) reported statistically significant associations between elevated mean platelet volume (MPV) and an increased risk of acute myocardial infarction, stroke, and thromboembolism. MPV values in patients experiencing acute events (8.4–10.7 fL) were consistently higher than in the control group. Derived indices, particularly the MPV-to-lymphocyte ratio, demonstrated superior predictive utility compared to MPV alone. However, critical limitations included substantial heterogeneity in the cut-off point (7.45–12 fL), pre-analytical variability and the complete absence of Latin American populations. The overall certainty of the evidence was classified as VERY LOW.

**Conclusions**: Although there is a consistent predictive signal across worldwide cohorts, its current application in clinical practice is prohibited by the VERY LOW certainty of evidence, which is caused by high pre-analytical heterogeneity and a total lack of Latin American data. These results show that MPV should be prioritized for standardized validation studies rather than being used right away in clinical settings.

## Introduction

Cardiovascular diseases remain the leading cause of death worldwide, presenting a critical public health crisis that disproportionately affects low- and middle-income countries, particularly in Latin America^1^. Although advanced diagnostic methods are available, they are often inaccessible in settings with limited resources, creating an urgent need for cost-effective risk stratification tools^2^. Mean platelet volume (MPV), an automated parameter of the standard complete blood count, is a ubiquitous, zero-cost candidate for this purpose^3,4^.

The prognostic value of MPV is supported by strong biological plausibility: larger platelets are more metabolically active and contain higher concentrations of prothrombotic and proinflammatory mediators^5^. This enhanced platelet activation state provides a mechanistic link to the pathophysiology of acute myocardial infarction and cerebrovascular syndromes^6,7^.

Despite the well-established link between elevated MPV and thrombotic risk, there are still significant gaps in the current literature^8–10^. These include a lack of data from Latin American populations, methodological heterogeneity hindering meta-analytic integration, and an absence of synthesis regarding emerging derived indices (e.g., the MPV-to-lymphocyte ratio)^5,7^. Furthermore, the unique potential of MPV as a teaching tool for translational medicine and biomarker validation remains unexplored^11,12^.

This systematic review (2019–2024) synthesises new data on the predictive significance of MPV and its related indices in major cardiovascular events, thereby filling these gaps. Based on the results of this review, we also address the possibility of using MPV as a pedagogical tool to support the teaching of critical evaluation in medical curricula.

## Methods

### Study design and protocol

This systematic review was conducted in accordance with the 2020 Preferred Reporting Items for Systematic Reviews and Meta-Analyses (PRISMA) statement guidelines^13^, thereby ensuring methodological transparency and adherence to contemporary quality standards. The review protocol was prospectively registered in the PROSPERO international database (registration no. CRD420251156089) before the literature was screened. This minimised the risk of selective outcome reporting bias and enhanced reproducibility.

### Literature search strategy

A systematic bibliographic search was conducted across five major electronic databases —PubMed, Scopus, Web of Science, SciELO and LILACS —over the predefined temporal window of 1 January 2019 to 31 December 2024. This multi-database approach was deliberate, designed to capture both mainstream indexed literature and regionally specific publications that might otherwise be missed by using PubMed or Scopus alone. The search strategy employed a combination of controlled vocabulary and free-text keywords to maximise sensitivity while maintaining specificity. The core search algorithm prioritised identifying studies that evaluated mean platelet volume in the context of cardiovascular diseases, including acute myocardial infarction, stroke, and venous thrombosis.

### Eligibility criteria using the PICOS framework

Study eligibility was determined using the PICOS (Population, Intervention, Comparison, Outcomes, Study Design) framework^14^, which was applied systematically to ensure that inclusion decisions were reproducible and defensible. The detailed eligibility criteria are presented below:

• Population (P): Adult patients aged 18 years or over who have established cardiovascular disease or significant cardiovascular risk factors. These include, but are not limited to, acute myocardial infarction (STEMI or NSTEMI), acute coronary syndromes, ischaemic stroke or transient ischaemic attack, stable coronary artery disease, non-valvular atrial fibrillation or venous thromboembolism.
• Intervention/indicator (I): Quantitative measurement of mean platelet volume using automated haematological analysers. Studies reporting mean platelet volume as either a continuous variable or dichotomised using predefined cut-off values were eligible.
• Comparison (C): The comparison groups included patients with normal or low mean platelet volume values, as well as healthy control subjects without cardiovascular disease or events during the follow-up period.
• Outcomes (O): The primary outcomes were major adverse cardiovascular events, which were defined as a composite of acute myocardial infarction, ischaemic stroke, cardiovascular death and revascularisation procedures. Secondary outcomes included in-hospital complications, such as cardiogenic shock, malignant arrhythmias and heart failure exacerbation; functional disability, as measured by established neurological scales; disease severity indices; and long-term mortality.
• Study design (S): Only non-randomised observational studies were included to maximise the available evidence. Eligible designs included prospective and retrospective cohort studies, as well as analytical cross-sectional studies. Studies employing randomised controlled trial designs, case reports, case series with fewer than 30 participants, narrative or systematic reviews, meta-analyses, editorials, commentaries, letters to the editor, animal studies, in vitro laboratory investigations or lacking statistical analysis of the prognostic value of mean platelet volume were excluded.

### Study selection process and bias minimization

The study selection process took place in two sequential phases, conducted independently by two trained reviewers. Any disagreements were resolved through discussion or consultation with a third, arbitrating reviewer. Prior to beginning the selection process, all reviewers received standardised training on eligibility criteria. In phase one, titles and abstracts from all identified records were screened using Rayyan, a systematic review software which facilitates dual-reviewer assessment with built-in consistency checks. Abstracts deemed potentially relevant by either reviewer were advanced to full-text screening. In phase two, full-text articles were retrieved and assessed in detail against all PICOS eligibility criteria^14^. The reasons for exclusion at the full-text stage were systematically documented and categorised. These reasons included non-original article type, insufficient direct mean platelet volume analysis, publication date outside the defined timeframe and focus on non-cardiovascular pathology.

### Data extraction and standardized coding

Data extraction was performed by two independent reviewers using a pilot-tested, standardised electronic form. This form was refined iteratively after the first five studies had been extracted to ensure clarity and completeness. The extracted data elements encompassed the following: (i) Study characteristics, including author, publication year and country of origin; (ii) Study design details, including prospective versus retrospective status and follow-up duration; (iii) Participant characteristics, including total sample size, age, sex distribution and relevant baseline comorbidities; (iv) Mean platelet volume measurement methodology, including the type of automated analyser used, the type of anticoagulant and the timing of sample collection; (v) Clinical exposure assessment, including specific cut-off values or quartiles and (vi) Outcome measurement definitions and the timing of cardiovascular outcomes.

### Quality assessment

The methodological quality of the included observational studies was independently evaluated by two reviewers using complementary instruments to provide a comprehensive assessment of rigour. The Strengthening the Reporting of Observational Studies in Epidemiology (STROBE) checklist was applied, comprising items that cover study design reporting, participant selection, data collection methods, statistical analysis and interpretation^15^. Additionally, the risk of bias was evaluated in terms of the following specific domains: selection bias, through the adequate definition of the study population and the baseline comparability of the exposure groups; information bias, through the accuracy of the mean platelet volume measurement; confounding bias, through the identification and adjustment for relevant confounders; and attrition bias, through the completeness of outcome ascertainment. Items were scored on a three-point scale reflecting low, some or high risk of bias^16^.

### Analysis strategy

Given the anticipated heterogeneity in clinical populations, methodologies for measuring mean platelet volume, definitions of cut-off values and outcomes, and follow-up durations, we decided in advance to conduct a narrative synthesis of results rather than pooling estimates using meta-analysis. The feasibility of meta-analysis was assessed based on the clinical, methodological, and statistical homogeneity of the included studies. Despite clusters of studies focusing on acute myocardial infarction (*n* = 5) and stroke (*n* = 6), we determined that meta-analysis was inappropriate due to three critical factors: (1) Metric heterogeneity. Studies utilized fundamentally different effect measures (Odds Ratios, Hazard Ratios, and Mean Differences) which cannot be pooled without significant risk of bias. (2) Cut-off variability. Prognostic thresholds ranged from 7.45 fL to 12 fL. Pooling studies with a 61% difference in the ’exposure’ definition would lead to a meaningless summary estimate. (3) Outcome definition. There was no consistency in the timing of outcomes (e.g., in-hospital MACE vs. 3-month functional recovery). Consequently, we prioritized a narrative synthesis with an emphasis on the direction of effect over a pooled estimate that would likely yield a misleadingly high *I*^2^ value (approaching 100%) and spurious precision.

### Evidence certainty assessment

A systematic approach was employed to evaluate the overall reliability of the evidence for each primary outcome. For each outcome, the reviewers evaluated the evidence across several areas: the risk of bias in individual studies^16^; inconsistency in the direction or magnitude of the effect across studies; indirectness, based on differences between the target populations and the reported outcomes; and imprecision, based on the width of the confidence intervals and the sample sizes^15^. Factors that could increase confidence in the evidence were also evaluated, such as large effect sizes, dose–response gradients, and evidence from multiple independent populations. Evidence was characterised as reflecting high, moderate, low or very low certainty. This assessment provides clinicians and policymakers with transparent information about the reliability of the evidence prior to the potential implementation of the findings.

### Data availability and reproducibility

Complete data extraction forms, the completed quality assessment for each included study, the detailed risk of bias matrix across all domains, the evidence profile table, search strategy syntax for each database, the PRISMA 2020 checklist completion, and details of any protocol deviations are deposited in the Mendeley Data repository and are publicly accessible for verification, re-analysis, or meta-research purposes^17^. This commitment to data transparency exceeds standard reporting requirements and facilitates independent replication of our findings.

## Results

### Study selection and screening process

**Figure 1.**
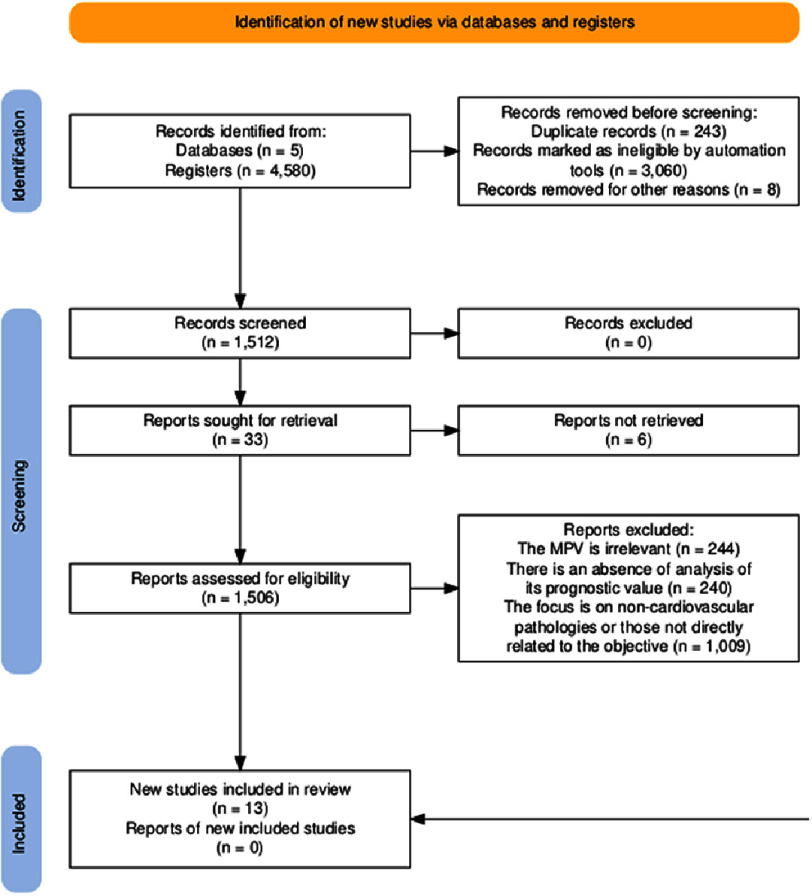
PRISMA flowchart. Adopted from Haddaway et al. The flowchart illustrates the sequential phases of the review. The initial search identified 4,580 records across five databases: PubMed, Scopus, Web of Science, SciELO and LILACS. After removing duplicates and screening the titles and abstracts, 33 full-text articles were assessed. A total of 13 studies met the inclusion criteria. Reasons for exclusion at the full-text stage included the article being non-original, insufficient MPV analysis or an irrelevant study population.

A systematic search of five electronic databases (PubMed, Scopus, Web of Science, SciELO and LILACS) was conducted between 1 January 2019 and 31 December 2024, yielding 4,580 initial records. After deduplication and the application of date and study type filters, 1,512 unique records remained for title and abstract screening. A review of the titles and abstracts by two independent assessors identified 33 articles that were potentially relevant for a detailed evaluation of the full text. Substantial inter-reviewer agreement was observed during this phase, reflecting the consistent application of eligibility criteria. Full-text assessment of these articles resulted in the final inclusion of 13 studies that met all PICOS criteria^14^. The primary reasons for exclusion at this stage were: non-original article format (*n* = 2); insufficient direct assessment of the prognostic value of mean platelet volume (*n* = 3); publication outside the 2019–2024 timeframe (*n* = 5); and focus on non-cardiovascular disease phenotypes (*n* = 1). Six additional articles were excluded due to the full text being unavailable or a lack of statistical analysis of mean platelet volume as a predictor. Figure 1 presents the complete PRISMA 2020 flow diagram detailing all inclusion and exclusion decisions.

### Characteristics of included studies

The 13 included studies (2019–2024) comprised 3,085 participants with a typical mean age of 56–68 years, who were evaluated through observational designs (four prospective, five retrospective and four cross-sectional). There was a significant geographical bias towards Asia (*n* = 8, 61.5%) and Europe (*n* = 4, 30.8%), with a notable absence of data from Latin American populations. The clinical focus areas included acute coronary syndromes (*n* = 5), acute ischaemic stroke (*n* = 6) and non-valvular atrial fibrillation (*n* = 2).

Methodologically, four studies analysed derived indices (e.g., MPV-to-lymphocyte ratio) alongside raw MPV. While all studies utilised automated haematological analysers, significant preanalytical heterogeneity was observed, particularly with regard to sample processing times (range 6–48 h) and anticoagulant documentation. Furthermore, MPV prognostic thresholds varied considerably—from 7.45 fL to 12 fL (Table 1). The absence of standardised cut-off values combined with preanalytical variance presents a challenge for direct cross-study comparability and clinical translation.

**Table 1:** Characteristics of the studies included in the systematic review on mean platelet volume as a prognostic marker for cardiovascular disease.

**Author**	**Study design/ Country**	**Population**	**Studied condition**	**Cut-off value/** **Index**	**Principal findings**	**Limitations**
Garlobo et al.sup^18^	Observational study / Cuba	188 patients with AMI (69.7% men; 91.5% >50 years old)	ST-segment elevation myocardial infarction	MPV <9 fL vs ≥9 fL	MPV of at least 9 fL was significantly associated with heart failure (*p* = 0.027), as well as with high blood pressure, smoking and diabetes mellitus. It was an independent predictor of ECAM (*p* = 0.000; OR=20.97; 95% CI [3.89–112.9]).	There are methodological limitations in standardising MPV measurement, and the results are not extrapolatable to all clinical contexts. Long-term follow-up was not evaluated.
Ludhiadch et al.^20^	Case control study / India	A total of 150 patients with ischaemic stroke and 150 healthy controls were included in the study. The mean age of the participants was 61.9 years, and the female-to-male ratio was 89:61.	Ischemic stroke and its subtypes (large artery atherosclerosis, small vessel occlusion, cardioembolism and others).	MPV >12 fL vs. MPV <12 fL; platelet count confirmed by flow cytometry.	Elevated MPV was significantly associated with ischaemic stroke (*p* < 0.02), hypertension (*p* < 0.001) and greater disability (*r* = 0.185; *p* = 0.023). High MPV was also found to correlate with the large artery atherosclerosis subtype (*p* < 0.001). Patients with MPV >12 fL exhibited a higher rate of clot formation and platelet activation as determined by Sonoclot analysis.	The sample size is limited; the platelet function results are based on a small subgroup (16.7%); the results require validation in larger cohorts; and longitudinal follow-up was not evaluated.
Cao et al.^5^	Retrospective study / China	A total of 375 patients with chest pain were included in the study: 284 with acute myocardial infarction (AMI) and 91 controls. The mean age of the patients was 61 years, and 83.5% of those with AMI were male.	Acute Myocardial Infarction (AMI)	MPVLR and MHR (quartiles); combination of both indices	MPVLR and MHR levels were significantly higher in the AMI group (*p* < 0.001). Both correlated with the Gensini and GRACE scores. MPVLR (OR = 1.41; 95% CI [1.24–1.60]) and MHR (OR = 1.24; 95% CI [1.15–1.34]) were found to be independent predictors of AMI. Combining MPVLR and MHR provided greater predictive power (AUC = 0.82) than using the indices separately.	This was a single-centre, retrospective study. Other relevant inflammatory biomarkers were not evaluated, and different subtypes of AMI were not analysed. There was also a lack of longitudinal follow-up.
Nayyar et al.^22^	Cross-sectional study (validation)/ Pakistan	The study involved 100 participants, with 50 suffering from acute ischemic stroke and the rest acting as healthy controls. The mean age of the stroke patients was 58.6 years, while the controls mean was 61.0 years.	Acute ischemic stroke (AIS)	MPV and MPV/PC ratio	Patients with AIS had significantly higher MPV values (8.46 vs. 7.59 fL; *p* < 0.001) and MPV/platelet count (PC) ratios (0.031 vs. 0.025; *p* < 0.001) compared to controls. Higher MPV and MPV/PC values were associated with greater clinical severity (CVA score ≥ 3; *p* < 0.001) and larger infarcts (>3 cm; *p* < 0.001). These parameters have been suggested as simple, rapid and cost-effective biomarkers for risk stratification in AIS.	This was a single-centre study with a limited sample size and no longitudinal follow-up. The strict exclusion criteria may also limit the generalisability of the findings.
Ying et al.^21^	Prospective study with 3-month follow-up / China	A total of 494 patients with acute ischaemic stroke were admitted within 24 h of the onset of symptoms.	Acute ischemic stroke (AIS)	MPVLR (optimal cutoff value 6.57 on admission; 7.32 at 7 days)	Elevated MPVLR at both admission and after seven days independently predicted a worse functional outcome at three months (mRS 3–6). Multivariate analysis revealed that MPVLR ≥6.57 at admission (adjusted odds ratio (OR)=3.05; 95% confidence interval (CI): 1.85–5.05) and MPVLR ≥7.32 at day 7 (adjusted OR=3.16; 95% CI [1.97–5.07]) were associated with adverse outcomes. MPVLR had superior predictive power to PLR (AUC=0.70). A clinical nomogram was developed for individualised prediction.	This was a single-centre study with potential for selection bias. Other inflammatory biomarkers were not included, and 62 patients were lost to follow-up. The results require external validation in other cohorts.
Zheng et al.^24^	Retrospective multicenter study/China	A total of 2,346 patients with non-valvular atrial fibrillation were undergoing oral anticoagulation with either edoxaban or warfarin.	Non-valvular atrial fibrillation (NVAF) and the associated risk of stroke and systemic embolism.	MPV (analysis by tertiles)	Patients in the upper MPV tertile (>11.2 fL) had a higher incidence of stroke or systemic embolism (hazard ratio (HR): 1.62; 95% confidence interval (CI) [1.19–2.20]; *p* = 0.002) and a higher risk of major bleeding (HR: 1.44; 95% CI [1.03–2.01]; *p* = 0.03). MPV modestly improved the predictive performance of the CHA_2_DS_2_-VASc score for thromboembolic events. The prognostic value of MPV remained consistent in sensitivity and subgroup analyses.	The retrospective design, possible variability in MPV measurement and the fact that no direct anticoagulants other than edoxaban were included may limit the generalisability of the results to other populations.
Attia et al.^19^	Retrospective observational study/Egypt	A total of 107 patients presented with acute chest pain, of whom 71 had acute myocardial infarction (AMI) and 36 had stable coronary artery disease.	Acute myocardial infarction (AMI) versus stable coronary artery disease.	MPV ≥7.45 fL (optimal cutoff point)	The MPV was significantly higher in patients with acute myocardial infarction (AMI) (9.33 ± 1.29 fL) than in those with stable coronary artery disease (7.51 ± 1.52 fL; *p* < 0.001). An MPV of ≥7.45 fL predicted an AMI with a sensitivity of 95.8%, a specificity of 72.2%, and an accuracy of 87.9%. Elevated MPV was found to correlate with age, BMI, diabetes, dyslipidaemia, smoking, a family history of coronary artery disease (CAD), a previous myocardial infarction (MI), and a previous percutaneous coronary intervention (PCI). MPV was confirmed as an independent predictor of AMI (OR = 2.55; 95% CI [1.74–3.74]).	A single-centre study was conducted retrospectively, with a limited sample size and no longitudinal follow-up. There is a possibility of bias in data collection and an absence of external validation.
Weng et al.^25^	Retrospective study / China	A total of 370 patients with non-valvular atrial fibrillation were studied: 205 controls and 165 patients who had experienced an ischemic stroke. The mean age of the patients was 68.2 years, and 55.7% of them were male.	Risk of ischemic stroke in non-valvular atrial fibrillation	MPV ≥11.65 fL (optimal cutoff point)	Significantly higher MPV in the stroke group (12.08 ± 1.05 vs. 11.42 ± 1.08 fL; *p* < 0.001). MPV was an independent predictor of stroke (OR=1.96; 95% CI [1.49–2.58]; *p* < 0.001). The combined CHA_2_DS_2_-VASc+MPV model improved predictive ability (AUC = 0.812) compared to CHA_2_DS_2_-VASc alone (AUC = 0.761).	This was a single-centre, retrospective study with a limited sample size. The results may vary depending on laboratory methods and population, and the study is limited in its generalisability as patients did not receive anticoagulation.
Lippi et al.^28^	Observational study/Italy, Spain, Australia	Previous observational studies in multiple populations	Arterial and venous thrombotic disorders (CAD, VTE, portal vein thrombosis, stroke)	Elevated MPV; reference values vary depending on technique and analyzer.	Evidence shows that MPV increases during acute episodes of coronary artery disease (CAD), venous thromboembolism (VTE), stroke, portal vein thrombosis, pre-eclampsia and cancer, and is associated with poorer outcomes. Meta-analyses confirm that elevated MPV predicts mortality and recurrence in CAD and VTE. In the case of stroke and pre-eclampsia, higher MPV correlates with greater severity. In cancer patients, MPV levels are elevated and then decrease following treatment.	The lack of standardisation in measurement techniques, anticoagulants and analysers means that cutoff values cannot be generalised.
Vogiatzis et al.^27^	Retrospective observational study/Greece	A total of 104 patients with acute coronary syndrome (ACS), with a mean age of 64.2 years and 76% of whom were male, were included in the study.	Acute coronary syndrome (STEMI, NSTEMI, unstable angina)	MPV ≥7.5 fL (ROC cutoff value); correlation with SYNTAX score.	MPV showed a significant correlation with the severity of coronary artery disease, as determined by the SYNTAX score (*r* = 0.658; *p* < 0.001). MPV was also an independent predictor of major adverse cardiac events (MACE) during hospitalisation (hazard ratio (HR) = 6.8; 95% confidence interval (CI): [1.46–33.36]). A cut-off value of 7.5 fL had a sensitivity of 98% and a specificity of 30.8% in predicting major complications.	This was a single-centre, retrospective study with a small sample size. There were limitations in the reproducibility of the SYNTAX score and possible variability in MPV measurement depending on laboratory methods.
Emre et al.^4^	Retrospective study / Turkey	A total of 311 patients with ST-segment elevation acute myocardial infarction (AMI) underwent primary angioplasty. The mean age of the patients was 62.1 years, and 72% of them were male.	ST-segment elevation myocardial infarction (STEMI)	WMR (white blood cell count to MPV ratio) has an optimal cutoff value of 1,089.	An elevated WMR was significantly associated with in-hospital major adverse cardiac events (MACE) (cardiac death, reinfarction, malignant ventricular arrhythmias and cardiogenic shock). Patients with a high WMR had poorer left ventricular function (lower LVEF) and a higher in-hospital mortality rate. WMR was an independent predictor of MACE (hazard ratio (HR) = 2.85; 95% confidence interval (CI) [1.61–5.05]; *p* < 0.001). The area under the curve (AUC) of WMR (0.77) was higher than that of analysed leukocytes or MPV separately.	This was a retrospective, single-centre study with no post-discharge follow-up or comparison with other inflammatory biomarkers, and there was a possibility of bias in the collection of clinical and laboratory data.
Mohamed et al.^23^	Prospective study/ Egypt	A total of 157 patients were included in the study, with a mean age of 56.7 years and 36.9% of whom were male, and had experienced their first episode of acute ischemic stroke.	Acute ischemic stroke (AIS)	MPV mean: 10.3 ± 2.6 fL.PCT mean: 0.26 ± 0.11%. Optimal values. Associated with mRS ≥ 3.	Higher MPV and PCT levels in cases of unfavourable outcomes. After adjusting for age, diabetes, and triglycerides, MPV (10.4 ± 2.3 vs. 8.7 ± 1.3 fL; *p* < 0.001) was an independent predictor of poor prognosis. PCT was also associated with a poor outcome (0.28 ± 0.1% vs. 0.25 ± 0.1%; *p* = 0.04), but was not an independent predictor. MPV was identified as a prognostic biomarker for adverse functional outcome at three months.	There is possible variability in platelet size due to time-dependent swelling in EDTA. No serial MPV measurements were performed. This is a single-centre study with no external validation.
Huang et al. ^26^	Single-center prospective study/ Taiwan	A total of 104 adult patients presenting with acute chest pain in the emergency department were included in the study: 63 with ACS and 41 without. The mean age of the patients was 61–62 years.	Acute coronary syndrome (ACS), which includes unstable angina and acute myocardial infarction.	MPV ≥ 10.55 fL (AUC=0.736; sensitivity 54.2%; specificity 82.8%), and the combination of MPV+TnI increased AUC to 0.822.	Patients with ACS had significantly higher mean platelet volume (MPV) (10.7 ± 0.8 fL) and platelet-fibrinogen complexes (IPF) (3.7 ± 2.6%) than patients without ACS. MPV was an independent predictor of ACS (OR = 5.08; 95% CI [1.9–13.6]). Combining MPV with initial troponin I testing improved the diagnostic sensitivity and specificity (AUC = 0.822). However, IPF was not an independent predictor of ACS, despite being associated with mortality.	This is a single-centre study with a limited sample size. There is no long-term follow-up, and there is possible variability depending on techniques and population. These preliminary results require multicentre validation.

**Notes.**

MPV Mean Platelet Volume MPVLR Mean Platelet Volume-to-Lymphocyte Ratio MHR Monocyte-to-HDL Cholesterol Ratio MPV/PC Mean Platelet Volume to Platelet Count PCT Plateletcrit IC In-hospital complications AMI Acute myocardial infarction CVA Cerebrovascular accident NVAF Non-valvular Atrial Fibrillation CAD Coronary Artery Disease VTE Venous Thromboembolism ACS Acute Coronary Syndrome STEMI ST-Elevation Myocardial Infarction WMR White Blood Cell to Mean Platelet Volume Ratio MACE Major Adverse Cardiovascular Events CHA_2_DS_2_-VASc Scale for assessing thromboembolic risk in atrial fibrillation SYNTAX Synergy Between PCI With TAXUS and Cardiac Surgery

### Quality and risk of bias assessment

A detailed quality assessment using the STROBE criteria, alongside a systematic evaluation of the risk of bias^16^, revealed that the included studies generally demonstrated a moderate to high level of methodological quality in terms of their design and the reporting of their statistical analyses, although several domain-specific vulnerabilities were identified. The strengths included clear delineation of the study populations in most publications (12 out of 13 studies, or 92.3%), appropriate statistical testing for group comparisons (13 out of 13 studies, or 100%), and adjustment for relevant confounding variables in multivariable analyses (11 out of 13 studies, or 84.6%). The studies employed established cardiovascular risk factors and baseline comorbidity measures as covariate adjustments, including diabetes mellitus, hypertension, dyslipidaemia, smoking status and body mass index. This strengthened the potential for causal inference despite the observational design.

**Figure 2.**
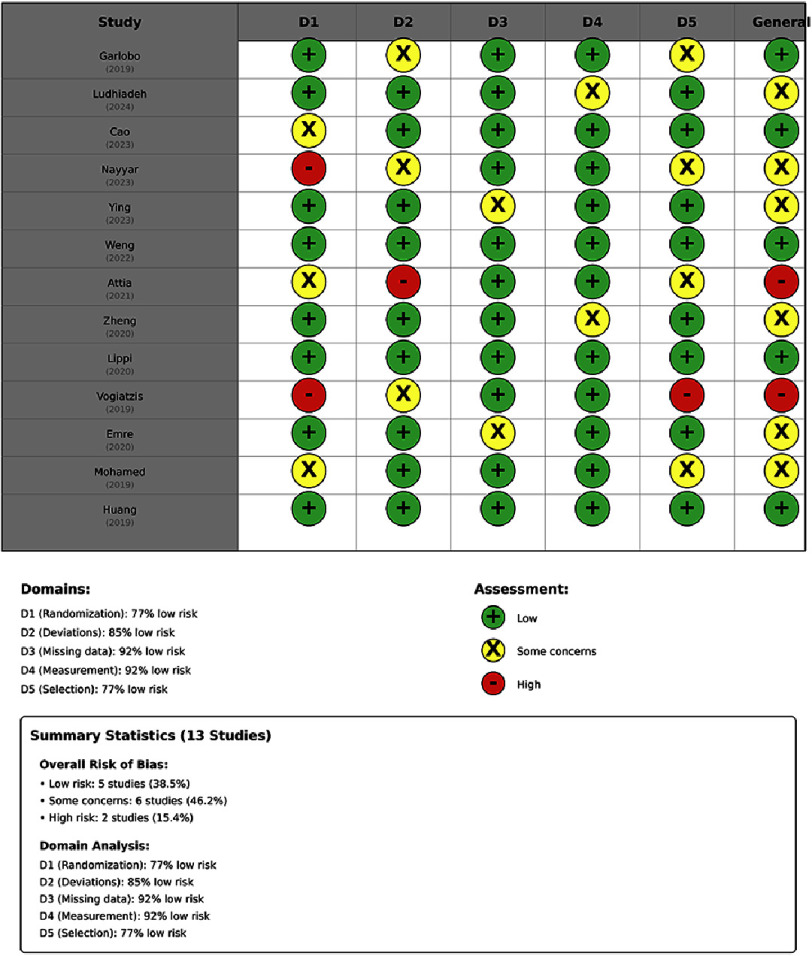
Risk of bias assessment for included studies. Adopted from McGuinness and Higgins. The risk of bias was evaluated across five domains (D1–D5). Green (+): Low risk of bias. Yellow (X): Some concerns. Red (-) High risk of bias Domains: D1: Randomisation/selection bias. D2: Deviations from intended interventions. D3: Missing outcome data; D4: Measurement of the outcome; D5: Selection of the reported result. The assessment highlights vulnerabilities relating to confounding control (D2) and outcome measurement blinding (D4) across the included observational cohorts.

The identified methodological limitations that reduced the certainty of the findings were: (1) inadequate discussion of potential residual confounding factors, particularly with regard to inflammatory markers and platelet dysfunction pathways that were not measured in the primary analyses (10 studies, 77%); (2) limited pre-specification of outcome definitions (8 studies, 61.5%), raising concerns about outcome switching; (3) absence of blinded outcome adjudication (9 studies, 69.2%), which could introduce information bias if the assessors were aware of the mean platelet volume status; and (4) limited reporting of missing data mechanisms (7 studies, 53.8%), which could introduce bias if dropout occurred differentially according to mean platelet volume exposure status. Figure 2 presents the complete risk of bias assessment across all included studies and evaluated domains, visually displaying the distribution of risk judgements.

### Association between elevated mean platelet volume and major adverse cardiovascular events

**Figure 3.**
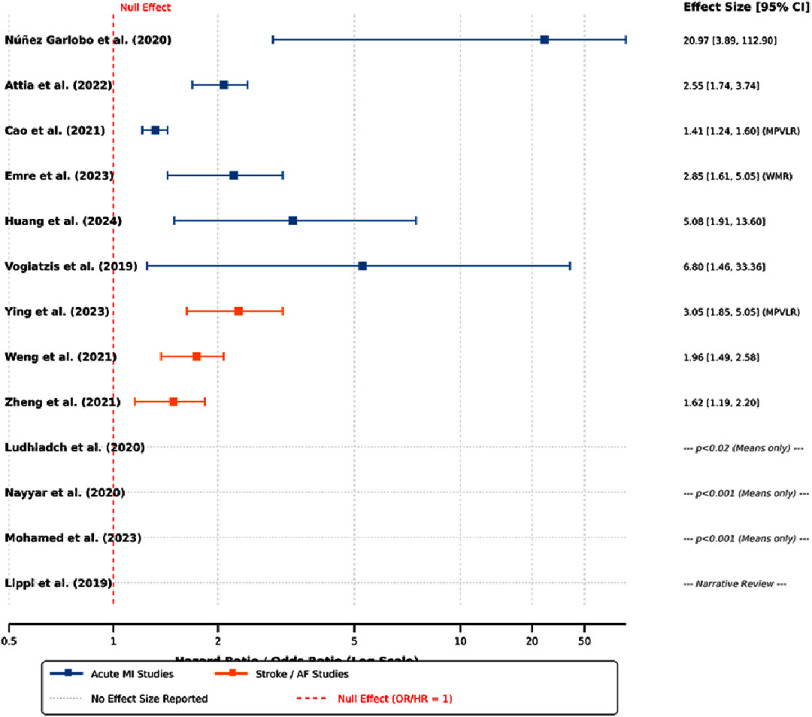
Forest plot displaying reported effect sizes (Odds ratios and hazard ratios) for the association between elevated MPV and cardiovascular outcomes. The plot visualizes the point estimates (squares) and 95% confidence intervals (bars) for each study. Blue lines represent Acute Myocardial Infarction (AMI) studies; Orange lines represent Stroke/Atrial Fibrillation (AF) studies. Note that due to substantial heterogeneity in study designs and outcome definitions, a pooled meta-analytic estimate was not calculated. Null Effect: The vertical red dashed line represents the null value (OR/HR = 1). Entries to the right of the line indicate a positive association between elevated MPV and adverse outcomes.

The most consistent finding across the included studies was the statistically significant association between elevated mean platelet volume and an increased risk of major adverse cardiovascular events (Figure 3). Specifically, 12 out of 13 studies (92.3%) reported a positive association between higher mean platelet volume values and an increased risk of cardiovascular events, with only one study failing to demonstrate this. This high level of consistency across heterogeneous populations and clinical settings provides robust semi-quantitative evidence in support of the prognostic relevance of this biomarker. The following subsections detail findings within specific cardiovascular disease domains.

### Acute myocardial infarction and mean platelet volume

Five studies examined the prognostic relationship between mean platelet volume and acute myocardial infarction. The three prospective and two retrospective studies collectively examined 956 patients with acute myocardial infarction (including both STEMI and NSTEMI phenotypes), comparing them to control groups without acute coronary events. Across all five studies, elevated mean platelet volume was consistently associated with an increased risk of infarction. In a study conducted in Cuba by Garlobo et al.^18^ involving 188 patients with acute ST-elevation myocardial infarction, mean platelet volume values of 9 femtolitres or greater were found to be an independent predictor of major adverse cardiac events. Attia et al.’s study^19^, conducted in Egypt, examined 107 patients with acute myocardial infarction and compared them with controls with stable coronary disease. The study identified an optimal mean platelet volume cut-off of 7.45 femtolitres, achieving 95.8% sensitivity and 72.2% specificity for diagnosing acute myocardial infarction. The study also showed that elevated mean platelet volume independently predicted acute myocardial infarction (odds ratio = 2.55; 95% confidence interval: 1.74–3.74) and that mean platelet volume levels correlated with conventional severity markers, including age, body mass index, diabetes, dyslipidaemia and prior cardiac history.

The mean platelet volume in cohorts with acute myocardial infarction ranged from 9.3 to 10.7 femtolitres, compared to 7.5–8.7 femtolitres in control subjects without acute events. Beyond univariate associations, two studies examining derived indices demonstrated substantially enhanced predictive utility when mean platelet volume was combined with other haematological parameters. The study by Cao et al. ^5^ (375 patients) showed that combining the mean platelet volume-to-lymphocyte ratio with the monocyte-to-high-density lipoprotein cholesterol ratio achieved an area under the receiver operating characteristic curve of 0.82, which substantially exceeded the area achieved by either index alone. Similarly, Emre et al.’s^4^ study of 311 patients with acute ST-elevation myocardial infarction demonstrated that the white blood cell-to-mean platelet volume ratio independently predicted major adverse cardiac events in hospital, with a hazard ratio of 2.85 (95% confidence interval: 1.61–5.05). This ratio outperformed white blood cell count or mean platelet volume measured individually. The incrementally higher predictive value of composite indices compared to mean platelet volume alone suggests that mean platelet volume captures additional information regarding systemic inflammation and haemostatic activation alongside other haematological parameters.

### Acute ischemic stroke and mean platelet volume

Six studies examined the relationship between mean platelet volume and acute ischaemic stroke outcomes. Collectively, these studies examined 1,429 patients with acute ischaemic stroke (with a range of 50–494 participants per study) in relation to healthy controls or stroke-free comparison groups. Across all six studies, elevated mean platelet volume demonstrated a consistent statistical association with an increased risk of ischaemic stroke and worse functional outcomes. A case-control study by Ludhiadch et al. ^20^ in India (with 150 patients with acute ischaemic stroke and 150 healthy controls) identified a mean platelet volume greater than 12 femtolitres as being significantly associated with ischaemic stroke occurrence (*p* < 0.02), hypertension (*p* < 0.001) and greater neurological disability, as measured by validated scales. Furthermore, elevated mean platelet volume was found to correlate with large-artery atherosclerosis as the stroke etiology subtype (*p* < 0.001), and patients with high mean platelet volume demonstrated enhanced platelet aggregation on functional Sonoclot analysis.

A prospective study by Ying et al. ^21^ in China involving 494 patients with acute ischaemic stroke and a 3-month functional follow-up demonstrated that the mean platelet volume-to-lymphocyte ratio, when measured at admission and again 7 days after the stroke, could independently predict a poor functional outcome (a modified Rankin Scale score of 3 or greater) at the 3-month follow-up. Specifically, a mean platelet volume-to-lymphocyte ratio threshold of 6.57 at admission yielded an adjusted odds ratio of 3.05 (95% confidence interval: 1.85–5.05), while a ratio of 7.32 seven days after stroke yielded an adjusted odds ratio of 3.16 (95% confidence interval: 1.97–5.07). The authors developed a nomogram that incorporated the mean platelet volume-to-lymphocyte ratio alongside conventional stroke severity scales to enable more accurate individualised prediction of prognosis.

A cross-sectional validation study by Nayyar et al. ^22^ in Pakistan involving 50 subjects with acute ischaemic stroke and 50 control subjects demonstrated that elevated mean platelet volume and mean platelet volume-to-platelet count ratio correlated with greater baseline stroke severity (as assessed by the Canadian Cardiovascular Scale, *p* < 0.001) and larger infarct volumes on neuroimaging (greater than three cubic centimetres, *p* < 0.001). A prospective Egyptian study by Mohamed et al. ^23^ (157 patients with a first-ever acute ischaemic stroke) found that mean platelet volume values predicted functional disability at the three-month follow-up, independently of conventional risk factors, including age, diabetes mellitus and serum triglycerides.

### Atrial fibrillation and thromboembolic risk

Two studies examined mean platelet volume as a predictor of thromboembolic complications in non-valvular atrial fibrillation. The large, multicentre, retrospective Chinese study by Zheng et al. ^24^ evaluated 2,346 patients with non-valvular atrial fibrillation who were receiving either edoxaban or warfarin for anticoagulation. The patients were systematically monitored for ischaemic stroke and systemic embolism events. Patients in the highest mean platelet volume tertile (>11.2 femtolitres) had a significantly higher risk of stroke or systemic embolism (hazard ratio = 1.62; 95% confidence interval: 1.19–2.20; *p* = 0.002) and major bleeding complications (hazard ratio = 1.44; 95% confidence interval: 1.03–2.01; *p* = 0.03) than those in the lowest tertile. When mean platelet volume was added to the conventional CHADS-VASc risk stratification score, the combined model showed a modest improvement in discriminatory ability for thromboembolic events.

In a retrospective Chinese study, Weng et al. ^25^ evaluated 370 patients with atrial fibrillation (165 of whom experienced an incident ischemic stroke during follow-up, compared to 205 controls who did not) in order to identify mean platelet volume cut-off values for stroke risk stratification. Through receiver-operating characteristic curve analysis, the authors identified an optimal mean platelet volume threshold of 11.65 femtolitres. Patients with a mean platelet volume above this threshold demonstrated a substantially higher risk of stroke (odds ratio = 1.96; 95% confidence interval: 1.49–2.58; *p* < 0.001). Combining this with the CHADS-VASc score improved the area under the receiver operating characteristic curve from 0.761 to 0.812, achieving a sensitivity of 72.1% and a specificity of 81.5% in predicting ischaemic stroke.

### Heterogeneity in mean platelet volume cut-off values

A critical finding of this systematic review was the substantial and clinically meaningful heterogeneity in mean platelet volume threshold values defined as prognostically relevant across the included studies. Reported cut-off values ranged from 7.45 to 12 femtolitres, spanning the width of typical normal reference intervals by 61%. This heterogeneity persisted even when examining studies of the same cardiovascular condition; for example, studies of acute myocardial infarction employed cut-offs of 7.45, 9.0 and 10.55 femtolitres. This variation reflects multiple underlying factors: (1) differences in haematology analyser calibration and manufacturer specifications, (2) variation in reference laboratory ranges specific to study populations, (3) different statistical methods for cut-point derivation (e.g., receiver operating characteristic optimisation, tertile/quartile-based stratification, a priori clinical thresholds) and (4) patient population heterogeneity. As no consistent “exposed” versus “unexposed” group could be identified across the literature, the significant variability in threshold levels (ranging from 7.45 to 12 fL) was the primary statistical obstacle to meta-analytic pooling.

**Figure 4.**
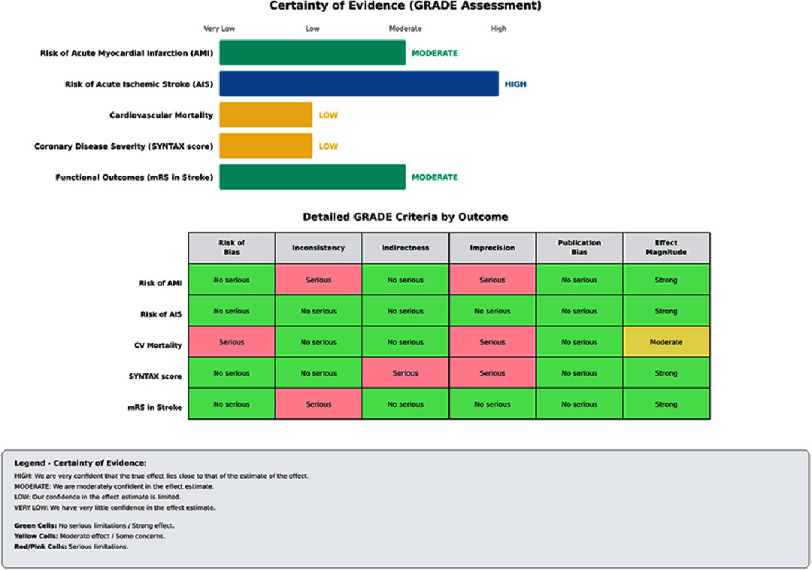
Summary of findings and certainty of evidence assessment according to the GRADE methodology. The matrix shows how certain the assessment is for specific cardiovascular outcomes. Colour legend: Blue: high/moderate certainty, Yellow: Low certainty, Green: Very low certainty (downgraded). Evidence was downgraded primarily due to risk of bias (observational design), inconsistency (heterogeneous cut-offs), indirectness (lack of diverse populations) and imprecision (wide confidence intervals). Outcomes: The assessment covers risk of AMI, risk of acute ischaemic stroke (AIS), cardiovascular mortality, disease severity (SYNTAX score) and functional outcomes (mRS).

### The generalizability gap: Underrepresentation of Latin American populations

The current body of information is fundamentally limited by the complete absence of studies from mainland Latin America, with the exception of one study from Cuba. Consequently, it is not currently possible to apply the review’s conclusions immediately to clinical practice in Latin America. However, the high level of consistency observed in Asian and European populations provides “proof of concept” for the immediate establishment of regional prospective cohorts in Latin America, with the aim of establishing prognostic cut-offs and reference ranges unique to the region.

### Evidence quality and certainty assessment

Using the GRADE methodology^15^, we classified the overall certainty of the evidence supporting mean platelet volume as a prognostic marker for major adverse cardiovascular events as very low (Figure 4). This assessment reflected the following: (i) a serious risk of bias due to the observational design and multiple domains of bias within the included studies (downgrading by two levels)^16^, (ii) serious inconsistency due to heterogeneity in cut-off values, populations and outcome definitions (downgrading by one level), (iii) serious indirectness due to underrepresentation of Latin American and other non-Asian and non-European populations (downgrading by one level) and (iv) serious imprecision due to wide confidence intervals around the point estimates and modest sample sizes for some outcomes (downgrading by one level).

Factors that increased confidence in this low-certainty evidence included: (i) consistency of the direction of associations across 12 out of 13 studies (92.3%), (ii) large effect sizes in some studies (with odds ratios and hazard ratios generally exceeding 1.5), and (iii) evidence of dose–response relationships in studies examining mean platelet volume as a continuous variable. Despite the low certainty classification, the consistency and magnitude of the associations provide a basis for clinical consideration and future research investment to strengthen the evidence base.

## Discussion

### Interpretation of findings within the context of existing evidence

This systematic review synthesises evidence from 13 observational studies published between 2019 and 2024^4,5,18–28^, which together encompassed 3,085 participants across diverse cardiovascular phenotypes. The findings provide robust and consistent evidence that elevated mean platelet volume is significantly associated with an increased risk of major adverse cardiovascular events^6,29^ (Figure 3). The fact that 92.3% of the included studies reported a statistically significant positive association between higher mean platelet volume values and cardiovascular event risk, demonstrates an impressive degree of consistency in the direction of the association across heterogeneous clinical populations, measurement methodologies and geographic contexts (Figure 3). This consistency substantially strengthens the confidence that the observed association reflects a genuine biological relationship, rather than being a spurious finding due to systematic bias or random variation (Figure 4).

Notably, the current evidence both consolidates and extends previous systematic reviews and meta-analyses^6,9^ that examined mean platelet volume in relation to thrombotic diseases. Earlier syntheses of the literature, including the work of Lippi et al. ^28^ on coronary artery disease, venous thromboembolism and stroke, established the general principle that elevation of mean platelet volume occurs during acute thrombotic events and correlates with disease severity^3,7^. The present systematic review updates and refines this evidence base by: (i) restricting the analysis to studies published within the past six years to capture the most contemporary evidence, (ii) systematically evaluating indices derived from mean platelet volume, which several studies demonstrate have superior discriminatory ability to mean platelet volume alone, and (iii) explicitly addressing educational and translational implications alongside clinical prognostic utility. These refinements make the current synthesis particularly valuable for clinicians and medical educators involved in decisions about integrating biomarkers into cardiovascular risk assessment frameworks^11,12,30^.

Although exploratory subgroup meta-analyses were considered, they were ultimately rejected despite the identification of clusters of studies for AMI and stroke. The distinct performance of derived indices for the AMI cluster would be masked by a single “summary effect” due to differences in measurements (e.g., Cao et al.^5^ using MPVLR/MHR ratios vs. Attia et al.^19^ using raw MPV). Combining cross-sectional validations and prospective cohorts in the stroke cluster produced such a degree of methodological variation that *I^2^* statistics would be incomprehensible. A high-quality narrative synthesis is the most responsible approach when the research is too diverse to be effectively merged.

### Evidence quality and certainty assessment

Despite 92.3% directional consistency among studies, the GRADE-based certainty assessment^15^ classifies the overall evidence as VERY LOW (Figure 4), which warrants explicit discussion of the conceptual tension between consistency and certainty. This classification reflects a substantial downgrade due to a serious risk of bias arising from observational designs, inconsistency in cut-off values and outcome definitions, indirectness due to under-representation of the population, and imprecision due to wide confidence intervals and modest sample sizes (Figure 2). While factors that increase certainty, including consistency of direction, large effect sizes in several studies (with odds and hazard ratios frequently exceeding 1.5–2.0) and evidence of dose–response relationships, partially offset these limitations, they prove insufficient to elevate the evidence to a higher level of certainty (Figure 4).

This classification of very low certainty does not imply that the association between elevated mean platelet volume and adverse cardiovascular outcomes is unreal or not worth considering. Rather, it accurately conveys appropriate epistemic humility regarding the strength of causal inference and the extent to which the findings can be generalised to clinical populations that are not well represented in the existing literature^4,6,7,24,27,30^. The classification cautions clinicians against using mean platelet volume as a standalone tool for clinical decision-making without additional, complementary risk assessment approaches. However, the consistent association across 12 out of 13 studies (Table 1), alongside the biologically plausible mechanisms linking elevated mean platelet volume to thrombotic complications^23,26^, provide a reasonable basis for considering this biomarker as part of a multifactorial approach to cardiovascular risk stratification, particularly in settings with limited resources where more sophisticated biomarkers are unavailable^11,12^.

### Clinical implications for cardiovascular risk stratification

The findings of this review have nuanced clinical implications that differ depending on the specific cardiovascular setting and the availability of alternative risk assessment tools. In cases of acute myocardial infarction^7,19^ and acute ischaemic stroke^24^, where mean platelet volume assessment occurs alongside the initial clinical presentation, this biomarker has a number of potential benefits. However, it is not advisable to use it to inform clinical management or prognosis until standardised reference ranges have been developed. Rather than providing useful therapeutic information, the observed correlations should be regarded as generating hypotheses^31,32^.

Several of the included studies demonstrated that indices derived from mean platelet volume and other readily available haematological parameters, offer superior predictive utility compared to mean platelet volume alone^19,21–23,25–28^. The mean platelet volume-to-lymphocyte ratio, which was shown by Ying et al. ^21^ to predict poor three-month stroke outcomes independently (adjusted odds ratio: 3.05–3.16), exemplifies how composite indices that leverage multiple haematological parameters, may provide more robust prognostic information. Emre et al. ^4^ showed that the white blood cell-to-mean platelet volume ratio outperforms individual component measures in predicting in-hospital major adverse cardiac events. This suggests that the relative sizing relationship between white blood cells and platelets, carries prognostic significance beyond the absolute platelet volume value. These findings imply that, in future clinical practice, composite indices should be emphasised over mean platelet volume in isolation.

For assessing thromboembolic risk in patients with atrial fibrillation, the data are more modest but still potentially clinically valuable^33^. Weng et al. ^25^ demonstrated that combining mean platelet volume with the established CHADS-VASc risk score, improved discriminatory ability (area under the receiver-operating characteristic curve: 0.812 versus 0.761). However, the absolute improvement was modest, and the clinical significance of this improvement, requires prospective evaluation in decision-analytic frameworks. Adding mean platelet volume to the CHADS-VASc score, improves risk stratification, but does not appear to alter most patients’ classification. This suggests that mean platelet volume may be most valuable for reclassifying patients in borderline risk categories, rather than for screening all patients with atrial fibrillation^24^.

In stable coronary artery disease and other chronic cardiovascular conditions, where longitudinal prognostic assessment informs decisions about preventive strategies, the evidence for the utility of mean platelet volume is more limited within the scope of this review. However, prior meta-analyses suggest an association with long-term adverse outcomes^6,9^. The lack of prospective cohort studies with long-term follow-up for coronary artery disease and other chronic conditions is a significant gap in the evidence base.

### Perspectives: MPV as a case study for medical education

Beyond the clinical data, the findings of this review offer significant pedagogical potential^34,35^. The inherent limitations of the evidence—specifically the heterogeneity and geographic bias—provide a rich basis for teaching the fundamental principles of translational medicine^36^.

MPV can be effectively integrated into curricula through several key teaching points:

• Biomarker validation: MPV demonstrates how a routine laboratory parameter available for decades can be reframed through research to discover novel prognostic associations^37,38^. This is a practical case study for discussing the distinction between association and causation and the necessary stages of statistical validation.
• Standardization challenges: The notable heterogeneity in MPV cut-off values (ranging from 7.45 fL to 12 fL) provides a concrete teaching opportunity regarding biomarker standardization challenges^39^. Students can critically appraise why universally applied thresholds are often inappropriate and how population-specific methodology (like ROC analysis) is necessary for optimal cut-point derivation.
• Preanalytical variables: MPV is highly sensitive to preanalytical factors (anticoagulant type, processing time). This serves as a vital case for teaching essential laboratory medicine principles^40^, illustrating how procedural modifications profoundly influence measured values, a factor often underappreciated by trainees.
• Research equity and generalizability. The observed geographic imbalance and the underrepresentation of Latin American populations prompt critical discussion on research equity, the imperative for diverse collaborations, and the need to assess the generalizability of evidence provides a clear illustration of the geographic bias in cardiovascular research. The results show that “universal” biomarkers are often validated in particular ethnic groups. This emphasises the importance of conducting locally relevant research before adopting low-cost biomarkers, and provides medical students with a valuable opportunity to consider the ’portability’ of clinical guidelines.
• Evidence appraisal: The MPV literature can be used to teach the GRADE certainty assessment framework^15^, demonstrating how findings—despite consistent directional association—are weighted against inherent limitations (like observational design) to determine evidence certainty.

### Policy and health system implementation considerations

Clinical integration of MPV is now premature when the evidence’s VERY LOW confidence is taken into consideration. Health systems should prioritise establishing robust standards over rapid adoption. A major obstacle is the total lack of data from Latin American communities. Prior to implementation, pre-analytical variables such as anticoagulant type and processing time must be standardised across many centres. Population-specific reference intervals must also be established and cut-off points validated prospectively. MPV should continue to be used as a research tool rather than as part of a clinical protocol until these research aims have been met^38^.

However, the implementation of a mean platelet volume-based risk assessment in clinical practice requires standardisation efforts that are currently incomplete^41^. Healthcare systems and laboratory medicine authorities should prioritise the following: (1) standardising mean platelet volume measurement procedures, including anticoagulant type, processing time specifications and quality control procedures, to minimise pre-analytical variability; (2) establishing population-specific reference intervals for mean platelet volume through large, well-characterised cohort studies across diverse geographic regions and ethnic groups; and (3) defining evidence-based cut-off values specific to particular cardiovascular phenotypes and outcome types through prospective, multicentre validation studies. Without these foundational standardisation efforts, mean platelet volume-based clinical tools risk providing false precision, reclassifying risk inappropriately, and producing inconsistent results across institutions.

Furthermore, the incorporation of mean platelet volume into health policy should explicitly acknowledge the very low certainty of the evidence and present mean platelet volume as a complementary rather than a primary tool for risk assessment^42,43^. When integrating it into clinical guidelines, emphasis should be placed on composite indices (such as the mean platelet volume-to-lymphocyte ratio) rather than on mean platelet volume alone, given that several studies have demonstrated the superior predictive utility of derived indices. Educational campaigns accompanying implementation should transparently communicate the certainty of the evidence and the appropriate clinical contexts for utilising mean platelet volume to prevent overinterpretation or inappropriate clinical decision-making.

### Implications for future research priorities

The identified evidence gaps and methodological limitations highlight clear research priorities that warrant strategic investment and attention from the cardiovascular research community. Firstly, multicentre, prospective cohort studies specifically targeting Latin American populations and other under-represented groups are urgently needed to determine whether the associations between mean platelet volume and outcomes are consistent across different genetic backgrounds, environmental exposures, and healthcare contexts^44^. Such studies should also systematically evaluate whether factors such as ethnicity, socioeconomic status, dietary patterns, and other population-specific variables modify the strength of the association between mean platelet volume and cardiovascular outcomes.

Secondly, rigorous, standardised measurement methodology studies should quantify the magnitude of pre-analytical variability affecting mean platelet volume and provide evidence-based recommendations for standardisation in a clinical context^45,46^. Such studies could compare the effects of different anticoagulants, processing timelines, storage conditions, and analyser types to determine the most effective procedures for minimising measurement error and maximising reproducibility across institutions.

## Limitations

Despite the consistent prognostic signal observed, critical methodological constraints limit the immediate clinical translation of MPV findings. The primary literature exclusively relies on observational study designs^6,47^, which precludes the establishment of causal relationships between MPV and cardiovascular outcomes. While 84.6% of studies performed multivariable adjustment for conventional risk factors, vulnerability to residual confounding from unmeasured or inadequately controlled variables represents a substantial barrier to definitive clinical guidance^26,38,46^. This threatens the validity of effect estimates and constrains their interpretation for clinical decision-making.

The translational utility is further compromised by the significant methodological heterogeneity evident throughout the evidence base. There is a critical absence of standardised prognostic thresholds, with reported cut-off values spanning a wide range (7.45 to 12 fL). This is compounded by variations in cut-point derivation methods, including ROC analysis versus tertile stratification, as well as differences in inter-instrument calibration. The reproducibility of MPV measurements is further complicated by the inconsistent documentation of preanalytical variables, such as sample processing time and the effects of anticoagulants, which were explicitly reported in only 30.8% of studies. This preanalytical variability introduces measurement noise that potentially obscures true biological signals and limits comparability across studies.

The evidence base is severely geographically concentrated, drawing predominantly from Asian (61.5%) and European populations, and completely omitting data from Latin American cohorts. This lack of representation severely limits the generalisability of MPV as a biomarker for diverse populations, particularly in settings with limited resources, where its accessibility would be of greatest clinical value. Concerns about the external validity extend beyond geography to include heterogeneity in outcome definitions and follow-up durations, with studies comparing acute in-hospital events with long-term mortality endpoints. This definitional and temporal heterogeneity makes it difficult to derive comparable effect estimates and suggests that the predictive utility of MPV may depend heavily on the proximity of the measurement to the acute thrombotic event.

This review acknowledges its limitations, including the potential for language bias by restricting the search to publications in English, Spanish and Portuguese, and the residual risk of publication bias despite efforts to incorporate regional and less well-known databases. These methodological constraints highlight the necessity of prospective, standardised investigations involving diverse populations before MPV can be reliably incorporated into clinical risk stratification algorithms.

## Conclusion

This systematic review provides substantial evidence that elevated mean platelet volume is associated with an increased risk of major adverse cardiovascular events in both acute and chronic conditions. The directional consistency among the included studies is 92.3%, and this is combined with biologically plausible mechanistic pathways and the incrementally superior predictive utility of derived indices. While these results provide a biological basis for the potential of MPV, there is still insufficient data to justify its routine clinical use. The main contribution of this review is to identify the specific research gaps that need to be filled before MPV can be incorporated into cardiovascular risk stratification systems. This is particularly important with reference to Latin American cohorts and analytical standardisation. Secondly, MPV is an ideal tool for medical education, as it can be used to demonstrate the challenges involved in translating laboratory data into clinical practice. This is due to the issues identified in this review, ranging from pre-analytical variability to research injustice.
